# NR4A Nuclear Receptors Target Poly-ADP-Ribosylated DNA-PKcs Protein to Promote DNA Repair

**DOI:** 10.1016/j.celrep.2019.01.083

**Published:** 2019-02-19

**Authors:** Deeksha Munnur, Joanna Somers, George Skalka, Ria Weston, Rebekah Jukes-Jones, Mohammed Bhogadia, Cyril Dominguez, Kelvin Cain, Ivan Ahel, Michal Malewicz

**Affiliations:** 1 MRC Toxicology Unit, Leicester LE1 7HB, UK; 2 Sir William Dunn School of Pathology, South Parks Road, University of Oxford, Oxford OX1 3RE, UK; 3 Centre for Biomedicine, Manchester Metropolitan University, Manchester M15 6BH, UK; 4 Department of Molecular and Cell Biology, University of Leicester, Leicester LE1 7RH, UK

**Keywords:** DNA repair, double-strand breaks, DSB, NHEJ, NR4A, DNA-PK, poly-ADP-ribose, PARP, non-homologous end joining, transcription factors

## Abstract

Although poly-ADP-ribosylation (PARylation) of DNA repair factors had been well documented, its role in the repair of DNA double-strand breaks (DSBs) is poorly understood. NR4A nuclear orphan receptors were previously linked to DSB repair; however, their function in the process remains elusive. Classically, NR4As function as transcription factors using a specialized tandem zinc-finger DNA-binding domain (DBD) for target gene induction. Here, we show that NR4A DBD is bi-functional and can bind poly-ADP-ribose (PAR) through a pocket localized in the second zinc finger. Separation-of-function mutants demonstrate that NR4A PAR binding, while dispensable for transcriptional activity, facilitates repair of radiation-induced DNA double-strand breaks in G1. Moreover, we define DNA-PKcs protein as a prominent target of ionizing radiation-induced PARylation. Mechanistically, NR4As function by directly targeting poly-ADP-ribosylated DNA-PKcs to facilitate its autophosphorylation-promoting DNA-PK kinase assembly at DNA lesions. Selective targeting of the PAR-binding pocket of NR4A presents an opportunity for cancer therapy.

## Highlights

• NR4A nuclear orphan receptors possess a poly-ADP-ribose (PAR)-binding pocket
• PAR binding is essential to NR4A activity in DNA repair
• Ionizing radiation induces PARylation of DNA-PKcs kinase
• NR4As promote DNA-PKcs autophosphorylation in a PAR-dependent fashion

## Graphical Abstract

**Figure:**
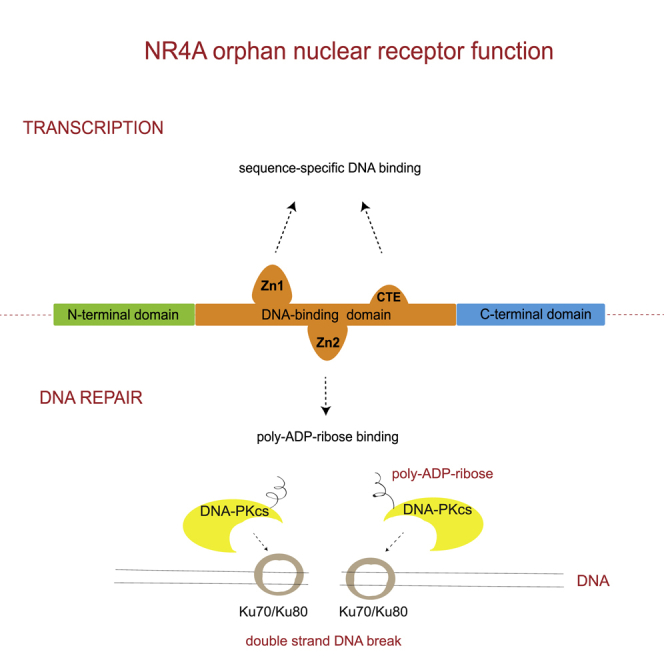

## Teaser

DNA damage induces poly-ADP-ribosylation (PARylation) of repair factors recruited to DNA lesions. Munnur et al. identify a zinc-finger-type poly-ADP-ribose-binding domain in NR4A nuclear orphan receptors that targets PARylated DNA-PKcs repair kinase, facilitating its autophosphorylation and repair of double-strand DNA breaks.

## Introduction

Nuclear DNA is under a constant threat of damage by reactive oxygen species, aberrant activity of enzymes and/or exogenous radiation. Therefore, efficient DNA repair is essential for survival of all organisms (Jackson and Bartek, 2009). Although double-strand breaks (DSBs) can be repaired by several pathways, classical non-homologous end joining (c-NHEJ) is responsible for the bulk of DSB repair (Waters et al., 2014). NHEJ is initiated by sequence-independent binding of Ku70/Ku80 dimers to exposed DNA ends at DSBs. DNA-bound Ku rapidly recruits DNA-PKcs kinase, resulting in its activation and DNA-PKcs autophosphorylation. Some DSBs undergo processing by Artemis nuclease before ligation of DNA ends catalyzed by the XRCC4/LigaseIV complex (Chiruvella et al., 2013). Posttranslational protein modifications have important regulatory function in DSB repair (Polo and Jackson, 2011). For example, protein poly-ADP-ribosylation (PARylation) catalyzed by poly-ADP-ribose polymerases (PARPs) (Crawford et al., 2018, Luo and Kraus, 2012) has been implicated in c-NHEJ (Luijsterburg et al., 2016) and can stabilize protein-protein interactions because of function of specialized poly-ADP-ribose (PAR)-binding domains (Ahel et al., 2008). The NR4A family of nuclear orphan receptors consists of three members in mammals (NR4A1–NR4A3) and one member in *Drosophila* (DHR38). NR4As are sequence-specific DNA-binding transcription factors that regulate essential cellular processes such as cell growth, metabolism, and differentiation (Safe et al., 2016). NR4As possess a conserved DNA-binding domain (DBD) composed of two zinc fingers (Zn1 and Zn2). Typically, the Zn1 contacts DNA (Meinke and Sigler, 1999), while Zn2 is not involved in direct DNA binding. A direct role for NR4As has been discovered in DNA DSB repair, but the mechanism remains elusive (Jagirdar et al., 2013, Malewicz et al., 2011, Ramirez-Herrick et al., 2011, Smith et al., 2008, Yin et al., 2017). Here we show that NR4As’ DBD is functionally unique, because it is able to bind both DNA and PAR. PAR binding occurs through the Zn2 region and targets poly-ADP-ribosylated DNA-PKcs to facilitate activity of the DNA-PK repair complex during c-NHEJ. Altogether, we define a function of NR4As in DSB repair and propose a way for pharmacological targeting of NR4A in cancer therapy.

## Results

### Conserved Zn2 of NR4A2 Encodes a PAR-Binding Domain

**Figure 1:**
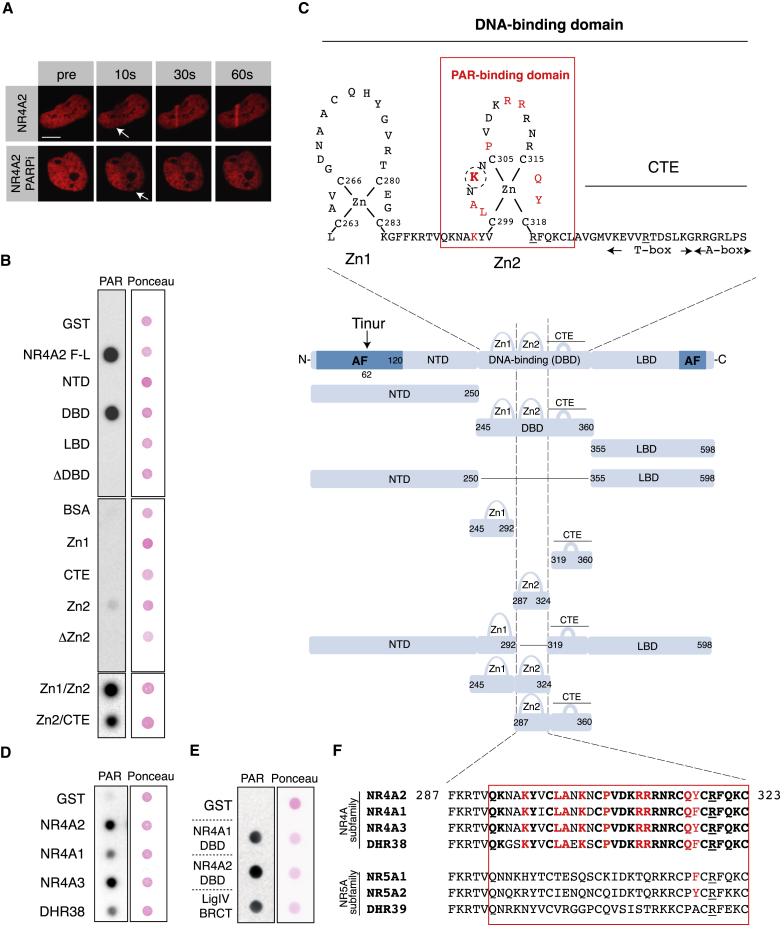
NR4A Nuclear Receptors Encode a Potent PAR-Binding Domain in Zn2 of Their DNA-Binding Domain (A) Laser microirradiation with transiently transfected mCherry-NR4A2 fusion proteins in cells pre-treated with DMSO vehicle or PARP inhibitor (PARPi). Arrow shows the irradiated position in the nucleus. Images have been taken at indicated time points after laser irradiation (s, seconds). Scale bar, 10 μm. (B) Radioactive poly-ADP-ribose (PAR) dot blot binding assays with recombinant proteins produced in *E. coli*. Ponceau S stain is used as protein loading control (Ponceau). (C) Schematic representation of the NR4A DNA-binding domain. Red box denotes the PAR-binding domain. Amino acids in red indicate residues involved in PAR binding; underlined residues are involved in DNA binding. Circled is the key residue involved in PAR binding. Middle panel shows a schematic representation of the NR4A protein structure. Vertical dashed lines show the position of the PAR-binding domain within the NR4A. F-L, full-length; NTD, N-terminal domain; DNA-binding (DBD), DNA-binding domain; LBD, ligand-binding domain; Zn1, zinc finger 1; Zn2, zinc finger 2; CTE, C-terminal extension; AF, transactivation domain. (D) Dot blot PAR-binding assays of full-length nuclear receptors from the NR4A family. (E) Dot blot PAR-binding assays of NR4A1 DBD, NR4A2 DBD, and LigaseIV BRCT domain. (F) Amino acid alignment of Zn2 domains. NR4A subfamily is depicted on top (DHR38 is *Drosophila* NR4A). Red frame denotes PAR-binding domain. Amino acids in bold indicate residues conserved within the NR4A subfamily. Amino acids in red indicate residues important for PAR binding. Amino acids underlined indicate residues important for DNA binding. NR5A family is shown in the bottom (DHR39 is the *Drosophila* NR5A).

Given that NR4A recruitment to DNA damage sites depends on PAR (Figure 1A) (Jagirdar et al., 2013, Malewicz et al., 2011), we asked whether NR4As could bind PAR directly. Recombinant NR4A2 protein strongly bound PAR (Figure 1B; Figures S1A–S1D). The ability to bind PAR resides in the second zinc finger (Zn2) of the NR4A2 DBD (Figures 1B and 1C). Although the isolated Zn2 region bound PAR weakly (Figure 1B, middle panel), addition of either Zn1 or C-terminal extension (CTE) restored PAR binding, suggesting that either Zn2 alone does not fold properly or Zn1 and CTE might contribute allosterically to Zn2 function (Figure 1B, lower panel). PAR-binding ability extends to all NR4A family members, including the *Drosophila* NR4A homolog DHR38 (Figure 1D). PAR and NR4A interaction is physiologically relevant, because it is comparable to that of the LigaseIV BRCT domain (Figure 1E), which binds PAR with nanomolar affinity (Li et al., 2013).

### DNA Binding and PAR Binding by NR4A2 Can Be Separated Biochemically

**Figure 2:**
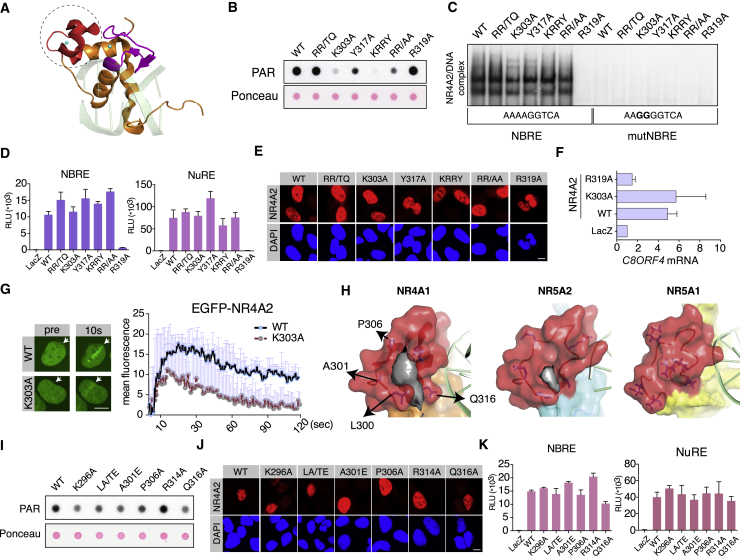
Generation of NR4A2 Separation-of-Function Mutants between PAR and DNA Binding and Identification of a PAR-Binding Pocket in NR4A Zn2 (A) Crystal structure of NR4A1 DBD with DNA oligonucleotide. Zn2 domain is shown in red and circled. Blue dots indicate zinc atoms. DNA is shown as a green shadow. (B) Dot blot PAR-binding assays of selected full-length NR4A2 mutants (RR/TQ, R310R312/TQ; KRRY, R310R311/TQ+K303A+Y317A; RR/AA, R310R311/AA). (C) Electrophoretic mobility shift assays (EMSAs) of full-length NR4A2 proteins showing sequence-specific DNA binding to NBRE or mutNBRE (mutated) oligo. (D) Reporter gene assays with NBRE- and NuRE-based luciferase reporters. RLU, relative luciferase activity. Graph represents mean RLU value (n = 3 with SD plotted). (E) Immunofluorescence of NR4A2 mutants after transient transfection into U2OS cells. DAPI indicates nucleus, red indicates NR4A2. Scale bar, 10 μm. (F) Induction of endogenous C8ORF4/TC-1 gene by transient transfection of NR4A2 into U2OS cells as measured by qPCR. Fold change over LacZ control is plotted (n = 3 with SD plotted). (G) Laser microirradiation of transiently transfected EGFP-NR4A2 fusion proteins. Arrow shows the irradiated position in the nucleus. Scale bar, 10 μm. Graph presents the quantification of laser microirradiation experiments (n = 3) with SD plotted above individual data points. (H) Comparison of Zn2 domains form NR4A1, NR5A1 and NR5A2 nuclear receptors with the putative PAR-binding pocket in gray. Arrows indicate mutated residues. (I) Dot blot PAR-binding assays with NR4A2 mutants (LA/TE, L300A301/TE). Ponceau indicates protein loading. (J) Immunofluorescence of NR4A2 mutants after transient transfection into U2OS cells. DAPI indicates nucleus, red indicates NR4A2. Scale bar, 10 μm. (K) Reporter gene assays with NBRE- and NuRE-based luciferase reporters. RLU, relative luciferase activity. Graph represents mean RLU value (n = 5 with SD plotted).

In the crystal structure of NR4A1 bound to DNA oligonucleotide (Meinke and Sigler, 1999), the Zn2 region is protruding away from DNA and does not contact DNA (Figure 2A). We thus assumed that separation of function between DNA binding and PAR binding would be possible. PAR recognition typically occurs via basic and aromatic amino acid residues (Ahel et al., 2008). We have generated various point mutants in full-length NR4A2 and assayed for PAR binding (Figure 2B; Figure S2A). Several mutants with reduced affinity for PAR were found. Combining these mutations in a quadruple mutant (KRRY) showed almost complete absence of PAR binding. None of these mutants showed any effect on sequence-specific DNA binding (Figure 2C). In contrast, arginine residue 319 (R319), conserved across the whole NR family (Figure S1E), contacts DNA (Meinke and Sigler, 1999), and R319A mutation abolished DNA binding (Figure 2C; Figure S2B) without affecting PAR binding (Figure 2B). All mutants (except R319A) prominently induced NR4A-specific reporter genes (Figure 2D) and showed normal nuclear localization (Figure 2E). To exclude a possibility that our synthetic reporter experiments failed to account for promoter- or enhancer-specific effects we identified the cellular gene *C8ORF4/TC-1* inducible by wild-type (WT) NR4A2 (but not R319A mutant) (Figure 2F; Table S1). NR4A2 K303A also induced expression of *C8ORF4/TC-1* (Figure 2F). K303A mutation showed consistent activity across multiple assays; therefore, it was selected as the prototypical separation-of-function mutant for further functional experiments. Furthermore, K303A strongly attenuated recruitment of NR4A2 to laser-induced DNA damage *in vivo* (Figure 2G). We also found that mutations of the first (CEAA), the second (C305A), or both (CC/AA) zinc-finger domains affected DNA binding, reporter-based transcription and PAR binding (Figures S2D–S2F) while maintaining normal nuclear localization (Figure S2C). Cysteine mutation of Zn1 (CEAA) also affected PAR binding (Figure S2F), consistent with our prior observation that Zn1 facilitates Zn2 folding or contributes allosterically to Zn2 activity (Figure 1B, middle and lower panels). We conclude that zinc-finger cysteine mutants of NR4A produce a broad effect on DBD functionality.

### Zn2 of NR4A Harbors a Distinct PAR-Binding Pocket

Localization of the PAR-binding domain to the Zn2 of NR4A suggested the presence of a cleft accommodating PAR. Molecular modeling revealed the presence of a distinct pocket in the Zn2 domain of NR4A1 (Figure 2H). Zn2s from the related NR5A class of receptors do not possess a similar pocket (Figure 2H), consistent with a divergent protein sequence (Figure 1F). Systematic mutagenesis of amino acids forming the NR4A2 Zn2 pocket revealed that most of these mutants reduced PAR-binding capacity (Figure 2I) without affecting DNA binding (Figure 2K) and maintained nuclear localization (Figure 2J). Comparison of NR4A PAR-binding pocket to ribofuranosyladenosine (RFA)-bound APLF (Eustermann et al., 2010) revealed that the binding surface of NR4A1 also has a wide cleft-like fold, which could accommodate PAR chains similar to APLF (Figure S2G). In summary, molecular modeling and empirical data identified a distinct PAR-binding pocket in the Zn2 domain of NR4A, which could potentially be targeted with NR4A-specific small molecules.

### NR4A1 and NR4A2 Redundantly Function in the c-NHEJ DSB Repair Pathway

**Figure 3:**
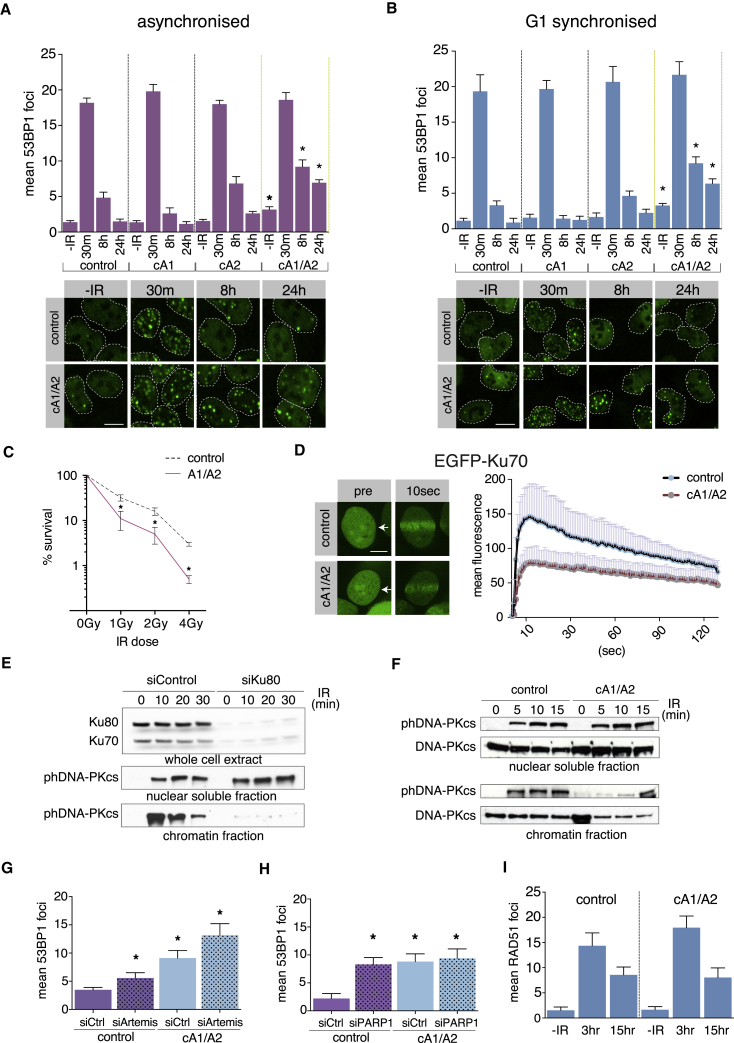
NR4A1/2 Nuclear Receptors Promote DSB Repair in G1 (A and B) DSB repair kinetics measured by 53BP1 foci resolution in asynchronized (A) or G1 synchronized (B) NR4A1/2 CRISPR cell lines at indicated time points (m, minutes; h, hours) after 1 Gy radiation. Graph shows mean foci value per nucleus with SEM plotted (n = 3). Asterisks denote statistically significant differences in relation to corresponding control sample (p < 0.05). Control indicates the parental U2OS cell line, cA1 indicates NR4A1 knockout cells, cA2 indicates NR4A2 knockout cells, and cA1/A2 indicates NR4A1/2 double-knockout cells. Images under the graphs show representative nuclei samples (white dashed lines) at indicated time points, showing 53BP1 nuclear stain and DSB foci (green signal). Scale bar, 10 μm. (C) Clonogenic survival assays. Control indicates the parental U2OS cell line, and cA1/A2 indicates NR4A1/2 double-knockout cells. Graph shows relative cell survival at the indicated radiation dose (n = 3; SD plotted on the graph). Asterisks denote statistically significant differences in relation to the corresponding control sample (p < 0.05). (D) Laser microirradiation of EGFP-Ku70 fusion protein in U2OS (control) and NR4A1/2 double-knockout (cA1/A2) cells. Arrow shows the irradiated position in the nucleus. Scale bar, 10 μm. Graph presents the quantification of laser microirradiation experiments (n = 5) with SD plotted above individual data points. (E) Western blotting of Ku70, Ku80, and phosphor-Ser2056 DNA-PKcs (phDNA-PKcs) proteins in U2OS cells transfected with control (siControl) or Ku80-specific (siKu80) siRNAs at indicated time points after 10 Gy irradiation. Top panel shows whole-cell extracts to reveal the extent of Ku80 depletion. Middle panel shows the nuclear soluble fraction. Lower panel shows the chromatin fraction. (F) Western blotting of DNA-PKcs and phosphor-Ser2056 DNA-PKcs (phDNA-PKcs) in either control cells or NR4A1/2 double-knockout cells (cA1/A2) at indicated time points after 10 Gy irradiation. Top panel shows the nuclear soluble fraction. Lower panel shows the chromatin fraction. (G) DSB levels measured as 53BP1 foci count per nucleus after 24 h post-1 Gy irradiation in control versus NR4A1/2 knockouts (cA1/A2) 72 h after transfection with reference siRNA (siCtrl) or Artemis-specific siRNA (siArtemis) (n = 3; error bars represent SEM). Asterisks denote statistically significant differences in relation to the control (siCtrl) sample (p < 0.05). (H) DSB levels measured as 53BP1 foci count per nucleus after 24 h post-1 Gy irradiation in control versus NR4A1/2 knockouts (cA1/A2) 72 h after transfection with reference siRNA (siCtrl) or PARP1-specific siRNA (siPARP1) (n = 3; error bars represent SEM). Asterisks denote statistically significant differences in relation to the control (siCtrl) sample (p < 0.05). (I) RAD51 foci count per nucleus at indicated times after 5 Gy irradiation in control versus NR4A1/2 knockouts (cA1/A2) (n = 3; error bars represent SD).

Given that U2OS cells express NR4A1 and NR4A2 (Malewicz et al., 2011), we derived NR4A1 (cA1), NR4A2 (cA2), or combined NR4A1/2 (cA1/A2) knockout lines by CRISPR (Figures S3A–S3C). NR4A1/2 double-knockout cells had a defect in DSB resolution (Figure 3A) and showed substantial radiosensitivity (Figure 3C), while single knockouts repaired DNA efficiently. cA1/A2 cells synchronized in G1 also showed delayed DSB repair (Figure 3B), suggesting an impairment of c-NHEJ repair pathway. cA1/A2 cells had slightly elevated basal level of DSBs (Figures 3A and 3B). Foci counting experiments were corroborated with neutral COMET assays, revealing a DSB resolution defect in NR4A-depleted cells (Figure S3D). NHEJ is initiated by Ku70/Ku80 dimer binding to free DNA ends at DSBs (Gottlieb and Jackson, 1993). NR4A double-knockout cells showed a substantial defect in Ku70 mobilization to DNA damage sites (Figure 3D). The current model of NHEJ activation places DNA-PKcs recruitment and autophosphorylation downstream of Ku loading (Meek et al., 2008). Depletion of Ku80 protein by small interfering RNA (siRNA) led to a dramatic reduction of autophosphorylated DNA-PKcs (phDNA-PKcs) on chromatin (Figure 3E) without affecting the levels of nuclear soluble phDNA-PKcs. cA1/A2 cells also showed strongly decreased levels of phosphoDNA-PKcs on chromatin at early time points after irradiation, although the effect was less dramatic in comparison to Ku80-depleted cells (Figure 3F; Figure S3E). Phosphorylation of DNA-PKcs in the nuclear soluble compartment was unaffected by NR4A1/2 deficiency. We conclude that NR4A1 and NR4A2 are redundant in facilitating DNA repair via c-NHEJ and control the levels of phDNA-PKcs on chromatin. Autophosphorylated DNA-PKcs is critically important for activation of Artemis nuclease in c-NHEJ (Goodarzi et al., 2006). Thus, we assessed the activity of Artemis in both control and cA1/A2 cells. As reported previously (Riballo et al., 2004), siRNA-mediated depletion of Artemis delayed repair of a subset of DSBs (Figure 3G; Figure S3G). Combined NR4A1/2 and Artemis deficiency resulted in an additive effect on DSB repair, demonstrating that Artemis activity was not compromised by NR4A1/2 deletion (Figure 3G). NR4A loss was epistatic with PARP-1 depletion (Figure 3H; Figure S3H). In contrast to NHEJ defect, homologous recombination (HR)-associated RAD51 foci efficiently formed and resolved in cA1/A2 cells (Figure 3I). Observed DSB repair defect in cA1/A2 cells was confirmed by HR- and NHEJ-specific reporter cassette assays (Figure S3F).

### NR4A2 Zn2 Targets Poly-ADP-Ribosylated DNA-PKcs to Facilitate DSB Repair

**Figure 4:**
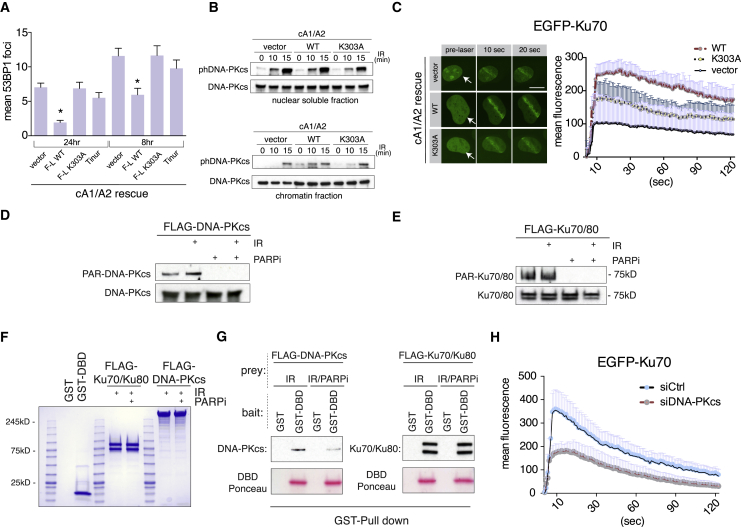
Zn2 PAR-Binding Domain of NR4A1/2 Facilitates DSB Repair by Targeting Poly-ADP-Ribosylated DNA-PKcs Protein (A) DSB levels measured as 53BP1 foci count after 8 or 24 h post-1 Gy irradiation in cA1/A2 after infection with recombinant retroviruses expressing indicated proteins (n = 3; error bars represent SEM). Asterisks denote statistically significant differences in relation to the vector control sample (p < 0.05). (B) Western blotting of DNA-PKcs and phosphor-Ser2056 DNA-PKcs (phDNA-PKcs) in cA1/A2 cells transduced with vector, WT NR4A2, or K303A NR4A2 expressing viruses at indicated time points after 10 Gy irradiation. Top panel shows the nuclear soluble fraction. Lower panel shows the chromatin fraction. (C) Laser microirradiation of transiently transfected EGFP-Ku70 fusion protein in cA1/A2 cells stably transduced with vector, WT NR4A2, or K303A NR4A2. Arrow shows the irradiated position in the nucleus. Scale bar, 10 μm. Graph presents the quantification of laser microirradiation experiments (n = 3) with SD plotted above individual data points. (D) Purified FLAG-DNA-PKcs protein from cells treated as indicated (PARPi, PARP inhibitor; IR, ionizing radiation, 20 Gy dose) were subject to western blotting with indicated antibodies. (E) As in (D) with purified FLAG-Ku70/Ku80 complex. (F) Coomassie staining of indicated protein preparations (glutathione S-transferase [GST]/GST-DBD NR4A2 shows these proteins after PreScission protease cleavage that releases DBD from GST). (G) GST pull-downs were performed with either GST or GST-DBD NR4A2 (aa 245–360)-bound beads (baits) incubated with indicated prey proteins. Bound material was eluted from GST beads by PreScission protease cleavage and either probed by western blotting for the presence of the DNA-PKcs/Ku complex or stained with Ponceau S to determine the efficiency of bait cleavage. (H) Laser microirradiation of transiently transfected EGFP-Ku70 fusion protein in U2OS cells pre-transfected for 48 h with control (siCtrl) or DNA-PKcs-specific siRNA (siDNA-PKcs). Graph presents the quantification of laser microirradiation experiments (n = 3) with SD plotted above individual data points.

To address the role of PAR binding by NR4A2 in DSB repair, we have re-expressed NR4A2 protein in cA1/A2 cells using the retroviral expression system (Figure S4A). Endogenous NR4A2 protein is expressed in U2OS cells as full-length receptor migrating at 66 kDa and a faster-migrating 60 kDa Tinur isoform (Okabe et al., 1995), which lacks 62 N-terminal residues (Figure 1C; Figures S3A and S4A). The Tinur isoform showed slightly diminished transcriptional activity in comparison to the full-length receptor (Figure S4B) even as it efficiently translocated to laser-induced DNA damage sites (Figure S4C). Unlike full-length WT NR4A2, Tinur failed to reverse the DSB repair defect in NR4A1/2 knockout cells (Figure 4A). NR4A2 K303A mutant also failed to rescue the DSB repair defect in cA1/A2 cells (Figure 4A; Figure S4D), although it showed a residual effect on DNA-PKcs autophosphorylation on chromatin and Ku70 recruitment to DNA lesions in cA1/A2 cells (Figures 4B and 4C). Furthermore, blocking cellular PARylation resulted in somewhat diminished Ku70 recruitment to DSBs (Figure S4E), consistent with earlier reports (Luijsterburg et al., 2016). In summary, full-length NR4A2 acts in c-NHEJ in a Zn2-dependent fashion.

We hypothesized that either DNA-PKcs or Ku complex might be the target of the NR4A2 Zn2 domain given that these proteins (but no other core NHEJ factors) had been reported to undergo PARylation *in vivo* (Martello et al., 2016) and both can interact with NR4A receptors (Malewicz et al., 2011, Zhao et al., 2011). We therefore purified DNA-PKcs protein and Ku70/Ku80 dimer to homogeneity and assessed their PARylation status (Figures 4D and 4E). Consistent with very high basal level of cellular PARylation (Figure S4F), we found PARylated DNA-PKcs and Ku in untreated cells (Figures 4D and 4E). Only PARylation levels of DNA-PKcs protein were elevated by ionizing radiation (IR) treatment (Figures 4D and 4E). Next, we compared the ability of DNA-PKcs/Ku proteins purified from IR- treated cells (maximal PARylation) against proteins purified from cells treated with IR and PARP inhibitor (no PARylation) to bind to the DBD of NR4A2. Although both DNA-PKcs and Ku bound the NR4A2 DBD, only DNA-PKcs showed PAR-dependent binding (Figure 4G). We next wondered of whether the diminished Ku70 recruitment observed in cA1/A2 cells was an indirect result of a defect in DNA-PKcs functionality. In striking similarity to cA1/A2 cells, depletion of DNA-PKcs protein in U2OS (Figure S4G) phenocopied the effect on Ku70 recruitment (Figure 4H). Thus, the assembly of the NHEJ complex in human cells is cooperative in the sense that the initial Ku binding leads to recruitment of DNA-PKcs, which in turn stabilizes Ku at DSBs. Ku stabilization is aided by PAR-dependent docking of NR4A2 to DNA-PKcs.

## Discussion

NR4As had primarily been recognized as sequence-specific DNA-binding transcriptional regulators (Safe et al., 2016). We and others proposed a direct role for NR4As in DNA DSB repair, which is a DNA sequence-independent process (Malewicz and Perlmann, 2014). In this manuscript, we present a model of how these contrasting activities of NR4As are executed. We find that DBD of NR4A is bi-functional and can bind DNA to regulate transcription (in a sequence-specific fashion) or PAR to facilitate DSB repair. Given that NR4A2 overexpression can accelerate DSB repair (Malewicz et al., 2011, Yin et al., 2017), it appears that the DNA-PK assembly is a rate-limiting step in this process. Thus, physiological and pathological conditions in which the expression of NR4A is altered, such as stress responses and various cancers, might be associated with variable NHEJ activity, with important consequences for cell physiology and drug responses. Loss of NR4A expression in acute myeloid leukemia (AML) is common and correlates with defective DSB repair (Ramirez-Herrick et al., 2011). In model organism *Dictyostelium*, the Ku70 homolog bears a PAR-binding domain, which is essential for effective c-NHEJ (Couto et al., 2011). Our data suggest that in mammalian cells, NR4As perform this accessory function. Many cancer therapies function by inducing persistent DNA damage (O’Connor, 2015). Inhibition of central NHEJ enzymes (DNA-PKcs and LigaseIV) was previously tested in the clinic, although these efforts were unsuccessful due to the toxicity of used compounds (Hosoya and Miyagawa, 2014). NR4As have long been perceived as attractive targets for drug discovery (Mohan et al., 2012). However, efforts to identify small molecules specific to NR4As were hampered by the lack of a suitable targeting strategy because of poor druggability of NR4A proteins (Wang et al., 2003). Our identification of a distinct PAR-binding pocket in Zn2 of NR4A, which operates in DSB repair, opens a way for development of NR4A2-specific small molecules for selective inhibition of NR4A2 PAR binding. These in principle could be of substantial value in cancer therapy, particularly on tumors characterized by prominent expression of NR4As, such as breast cancer (Aesoy et al., 2015, Llopis et al., 2013). We acknowledge that substantial work is necessary to realize this potential and to show benefit over currently used broad PARP inhibitors such as olaparib.

## STAR★Methods

### Key Resources Table

**Table:** 

REAGENT or RESOURCE	SOURCE	IDENTIFIER
**Antibodies**		

Mouse anti-tubulin	Sigma-Aldrich	Cat# T6074; RRID:AB_477582
Mouse anti-Ku70	Santa Cruz	Cat# sc-17789; RRID:AB_628454
Mouse anti-Ku80	Santa Cruz	Cat# sc-5280; RRID:AB_672929
Rabbit anti-phospho-DNA-PKcs ser2056	Abcam	Cat# ab18192; RRID:AB_869495
Mouse anti-DNA-PKcs	Abcam	clone 18-2; RRID:AB_731982
Rabbit Anti-NR4A1	BosterBio	Cat# PB9766
Rabbit Anti-NR4A2	Abcam	Cat# ab41917; RRID:AB_776887
Rabbit Anti-Artemis	CST	Cat# #13381
Rabbit anti-H2AX	Abcam	Cat# ab11175; RRID:AB_297814
Mouse anti-53BP1	Millipore	Cat# MAB3804; RRID:AB_2256673
Mouse anti-RanBP-1	Santa Cruz	Cat# sc-374352; RRID:AB_10990126
Rabbit anti-TBP	Santa Cruz	Cat# sc-204; RRID:AB_632480
Rabbit anti-PAR	Trevigen	Cat# 4336-APC-050; RRID:AB_10643399
Mouse anti-FLAG (M2)	Sigma-Aldrich	Cat# F3165; RRID:AB_259529
Rabbit anti-PARP-1	Abcam	Cat# ab32138; RRID:AB_777101
Rabbit anti-RAD51	Abcam	Cat# ab133534; RRID:AB_2722613
Rabbit anti-Ph-(Ser/Thr) PIKK substrate	CST	Cat# 2851S
Goat anti-mouse HRP conjugated	Pierce	Cat# 32430; RRID:AB_1185566
Goat anti-rabbit HRP conjugated	Pierce	Cat# 32460; RRID:AB_1185567
Goat Anti-Mouse IgG FITC conjugated	Jackson Immunores.	Cat# 115-095-146; RRID:AB_2338599

**Chemicals, Peptides, and Recombinant Proteins**		

Glutathione S-Sepharose 4B	GE Healthcare	Cat. 17075601
M2 agarose	Sigma-Aldrich	Cat. A2220; RRID:AB_10063035
^32^P-labeled NAD	Hartmann Analytic	Cat. ARP0141

**Critical Commercial Assays**		

Fast SYBR Green Master Mix	ThermoFisher	Cat. 4385612
Subcellular fractionation kit	ThermoFisher	Cat. 78840
Pierce Firefly Luciferase Glow Assay Kit	ThermoFisher	Cat. 16177
Galacto-Light Plus β-Galactosidase Assay	ThermoFisher	Cat. T1007

**Experimental Models: Cell Lines**		

U2OS	ATCC	Cat# HTB-96, RRID:CVCL_0042
293H	Invitrogen	N/A
293H stably expressing FLAG-DNA-PKcs	Craxton et al., 2015	N/A

**Oligonucleotides**		

*GAPDH* F GGAGTCAACGGATTTGGTCGTA	This study	N/A
*GAPDH* R GAATTTGCCATGGGTGGAAT	This study	N/A
*C8ORF4/TC-1* F AAGCCACCAAGCCATCATCA	This study	N/A
*C8ORF4/TC-1* R TCTTGGCTCTCTCCTCTGCT	This study	N/A

**Recombinant DNA**		

pGEX-6P-1	GE Healthcare	Cat. 28-9546-48
pCMX mammalian expression vector	Malewicz et al., 2011	N/A
pEGFP-N1	Clontech	N/A
pBabe-puro	Addgene	Cat. 1764
pDRGFP	Addgene	Cat. 26475
pimEJ5GFP	Addgene	Cat. 44026
pGL3-Basic	Promega	N/A

**Software and Algorithms**		

Prism 6 for MacOS X	GraphPad Software	N/A
PyMOL Molecular Graphic system, version 1.6.0.0	Schrödinger, LLC	N/A

### Contact for Reagent and Resource Sharing

Further information and request for resources and reagents should be directed to and will be fulfilled by the Lead Contact, Michal Malewicz (mzm23@mrc-tox.cam.ac.uk).

### Experimental Model and Subject Details

#### Cell culture

U2OS human osteosarcoma cell line was obtained from ATCC and cultured in DMEM medium (high glucose) supplemented with 10% FCS. 293H cells were purchased from Invitrogen and cultured in DMEM medium (high glucose plus pyruvate) supplemented with 10% FCS. For G1 cell cycle synchronization standard double thymidine block protocol had been used.

### Method Details

#### Western blotting

Cell extracts (WCE/whole cell extracts or nuclear extracts) were mixed with 4x Laemmli sample buffer (Bio-Rad; cat. 1610747) and boiled for 5min. Samples were then resolved on pre-cast TGX gradient 4%–15% gels (Bio-Rad). Gels were transferred to nitrocellulose membranes via Trans-Blot Turbo system (Bio-Rad) according to manufacturer’s instructions. After transfer, the membranes were blocked in BLOTTO TBS-T blocking solutions for at least 30min at room temperature. Primary antibodies were applied in 5% BSA TBS-T solution for overnight 4°C incubations. Secondary antibodies (HRP-coupled stabilized IgG from Pierce) were applied in TBS-T solution for 1hr at room temperature. Western blots were subsequently developed with Clarity HRP substrate (Bio-Rad) and exposed to Hyperfilm ECL films (GE Healthcare).

#### Plasmids/Cloning

GST fusion vectors were constructed by inserting PCR amplified full length NR4A1, NR4A2, NR4A3, DHR38, NTD/DBD/LBD/Zn1/Zn2/CTE of NR4A2 and DBD of NR4A1 coding fragments or hLigaseIV BRCT domain (aa 600-911) into pGEX-6P-1 vector (GE Healthcare) using the Quick ligation kit (NEB Inc). The PCR amplified inserts were prepared using the Q5 High–Fidelity DNA polymerase (NEB Inc). The inserts were cloned into the pGEX-6P-1 vector at the BamHI (or BglII) and XhoI cloning sites. Cloning was verified by diagnostic digestion using restriction enzymes followed by DNA sequencing (PNACL - University of Leicester, Leicester, UK). pCMX mammalian expression vectors encoding full-length NR4A1, NR4A2, NR4A3 and DHR38 were previously described (Malewicz et al., 2011). For the generation of mCherry-NR4A2 fusions PCR amplified full-length mouse NR4A2 were subcloned in mCherry-LacI plasmid (gift from Dr Tom Misteli, NIH, USA). For generation of EGFP-NR4A2 fusions PCR amplified full-length mouse NR4A2 was cloned in-frame into pEGFP-N1 plasmid (Clontech).

#### Generation of recombinant retroviruses

VSV-pseudotyped retroviruses were generated with Clontech Pantropic Retroviral Expression System (Cat. No. 631512) based on pBabe-puro (Addgene) vector and according to manufacturer’s instructions. Recombinant retroviruses carrying empty vector, F-L NR4A2 WT, Tinur or F-L NR4A2 K303 cDNAs were generated according to manufacturer’s instructions. In brief GP2-293 packaging cell line (Clontech/TaKaRa) was transiently transfected with a mixture of plasmids containing pBabe-puro vector and coat expression plasmid (VSV). After 72hr of incubation conditioned medium was harvested, filtered through 0,45 μm syringe top filter and applied to target cells in the presence of polybrene (SantaCruz). After 24hr of incubation stably transduced cells were selected out by puromycin selection (2 μg/ml; Invitrogen).

#### SiRNA-mediated mRNA knockdown

Control (reference), Ku80-, Artemis-, PARP1-, DNA-PKcs-specific siRNA were purchased from SantaCruz Biotechnology and transfected to U2OS by Lipofectamine RNAiMAX (Life Technologies) at 50nmol concentration. Cells were analyzed at 48-72hr post transfection.

#### Site directed mutagenesis

The site directed mutagenesis was performed using PfuUltra High Fidelity DNA polymerase (Agilent Technologies). The reaction mixture contained 0.2mM dNTPs, 50ng template plasmid, 2.5U Pfu DNA polymerase and 125ng of each primer. PCR amplification reaction required 18 cycles of step 1: 98°C for 30sec, step 2: 95°C for 30sec, step 3: 55°C for 1min, step 4: 68°C for 5mins. PCR product was subjected to 2 cycles of incubation with 20U DpnI (NEB Inc) enzyme at 37°C for 1h, then transformed into NEB 5-alpha *E.coli* strain (NEB Inc). Recovered plasmids were sequenced via in-house sequencing facility (PNACL - University of Leicester, Leicester, UK) to identify desired mutations.

#### Recombinant protein expression and purification

1-10ng/μl of pGEX-6P-1 plasmid containing desired cDNA for expression (GE Healthcare) was transformed in 10 μL aliquot of Rosetta 2 competent cells (Novagen). Transformed cells were spread onto ampicillin agar plate and incubated overnight at 37°C. Individual colony was picked into a 100mL LB starter culture containing 100 μg/mL ampicillin and 34 μg/mL chloramphenicol and incubated overnight at 37°C. The starter culture was further expanded into 500mL culture containing ampicillin and chloramphenicol and incubated for 6h at 37°C. The culture was then induced with 0.1mM IPTG and allowed to express the recombinant protein overnight at 18°C. Following induction, the culture was supplemented with 50 μM ZnCl_2_. Cells were harvested by centrifugation at 6000 g for 15mins at 4°C. The cells were suspended in 20mL GST lysis buffer (50mM Tris pH 7.5, 150mM NaCl, 1% Triton X-100, 0.1% β-mercaptoethanol, 0.4mM PMSF, 1X EDTA free Complete Protease Inhibitor cocktail (Roche) and 1mM benzamidine) and lysed by sonication. The lysate was then centrifuged at 20000 g for 30mins at 4°C. The cleared cell lysate supernatant was incubated with 200 μL pre-washed gluthathione Sepharose 4B (GE Healthcare) for 1h at room temperature. The protein bound beads were washed thoroughly with GST-wash buffer (50mM Tris pH 7.5, 500mM NaCl, 1% Triton X-100, 0.1% β-mercaptoethanol, 0.4mM PMSF, 0.5X EDTA free Complete Protease Inhibitor cocktail (Roche) and 1mM benzamidine). The beads were equilibrated with cleavage buffer (50mM Tris pH 7.0, 150mM NaCl and 1mM DTT). To obtain GST-tag cleaved recombinant proteins the beads were incubated with 100 μL cleavage buffer supplemented with 15U PreScission Protease (GE Healthcare) overnight at 4°C. Protein concentration was measured using NanoDrop 2000 UV-Vis Spectrophotometer (Thermo Scientific). For experiments shown in Figure 2B NR4A2 full-length protein preparations were further purified by gel filtration on Äktamicro system (GE Healthcare) using Superdex 200 Increase 3.2/300 gel filtration column according to manufacturer’s protocol. The proteins were further electrophoresed on precast 4%–15% Mini-PROTEAN TGX gels (Bio-Rad) and stained with quick Coomassie stain (InstantBlue; manufactured by Expedeon) to assess quality.

#### Neutral COMET assays

To induce double strand DNA breaks (DSBs) cells were treated with 100 μg/ml of zeocin (bleomycin derivative; Invitrogen) for 30min. Thereafter cells were either washed with medium to remove the zeocin and allow DNA repair to continue for 90min or harvested directly for COMETs. 100x10^3^ cells were mixed with 100 μL of LMP agarose in PBS (In-Cert, LONZA) and spread on a frosted microscope slide (precoated with standard 1% agarose; LONZA) and covered with 64mm coverslip. Agarose was allowed to solidify for 15min in the fridge. Subsequently coverslip was removed and the microspore slide with cells embedded in agarose was incubated in neutral lysis solution (2% sarkosyl, 0.5M EDTA, 0.5mg/ml proteinase K, pH 8.0) overnight in the fridge followed by 1hr at 37°C. Slides were then submerged in 1xTBE buffer for 30min in the cold room and subsequently electrophoresed at 15V for 15mins in 1xTBE buffer. Slides were recovered from the electrophoresis chamber and placed in DNA precipitation solution (1M ammonium acetate in 99% ethanol) for 30min at room temperature. Slides were then washed with 70% ethanol for 10min at room temperature, air-dried and stained with SYBRGOLD stain. Subsequently slides were mounted with Vectashield HardSet mounting medium (Vector Labs). COMETs images were acquired on a fluorescent microscope and quantified as percentage (%) of DNA in tail with CometScore (TriTec Corp., USA). At least 30 COMETs were scored per sample.

#### Purification of FLAG-DNA-PKcs and FLAG-Ku70/Ku80 proteins from mammalian cells

FLAG-tagged proteins were purified from suspension grown 293 cells (for FLAG-DNA-PKcs a stable clone was used; for FLAG-Ku70/Ku80 complex cells were transiently transfected with pCMX-FLAG-Ku70 and pCMX-FLAG-Ku80 expression vectors). Cells were washed twice in ice-cold PBS-MC (PBS, 1 mM MgCl_2_, 1 mM CaCl_2_) and gently resuspended in 4.5 ml ice-cold Hypotonic Buffer (10 mM HEPES, pH 7.9, 10 mM KCl, 0.1 mM EDTA, 0.1 mM EGTA supplemented with Complete protease inhibitors (Roche Diagnostics), 10 mM NaF, 1 mM Na3VO4, 10 μM MG132, 1 mM DTT, 1 mM PMSF). After incubation for 15 min, Igepal CA630 was added to 0.05% final concentration and cells were vortexed for 10 s and centrifuged at 2300 × g for 5 min. Crude nuclei were washed with 1 ml Hypotonic Buffer and re-centrifuged as above. Pellets mixed by end-to-end rotation with 15 ml high salt buffer (20 mM HEPES, pH 7.9, 500 mM NaCl, 0.5 mM MgCl_2_, 20% glycerol) supplemented as described above for 30 min on ice. Following centrifugation at 15000 × g for 30 min, nuclear extracts were incubated with M2 beads (SigmaAldrich) for 1-2hr on ice, beads were subsequently washed 3x10ml of 1M NaCl containing buffer to remove any associated proteins. Pure proteins eluted with elution buffer containing 0.2 mg/ml 3X FLAG peptide (SigmaAldrich).

#### GST pull downs

GST control protein (expressed from empty pGEX-6P plasmid) or GST-DBD NR4A2 (aa245-360 as indicated on Figure 1C) were expressed in bacteria according to standard GST purification protocol (see above) and bound to GST beads. Equimolar amounts of pure FLAG-DNA-PKcs (derived from a cell line stably expressing FLAG-DNA-PKcs protein) or FLAG-Ku70/Ku80 (derived from cells transiently transfected with pCMX-FLAG-Ku70/pCMX-FLAG-Ku80 plasmids) proteins purified from DMSO or PARPi (pan-PARP inhibitor - olaparib; 10 μM; pre-treatment 3hr prior to IR) pre-treated cells plus/minus 20Gy ionizing radiation (IR) were incubated with GST beads for 1hr on ice in a buffer containing 50mM Tris pH 7.5, 150mM NaCl, 0.5% Triton X-100 and 1mM DTT. Thereafter beads were washed with 3x1ml of binding buffer containing 300mM NaCl. Beads were equilibrated in PreScission protease cleavage buffer and then digested with 10U of PreScission protease in 75ul buffer volume for 4hrs. Eluates were run on SDS-PAGE and bound DNA-PKcs or Ku70/Ku80 were detected by western blotting. The efficiency of bait cleavage was confirmed by Ponceau S (Sigma) staining of the membrane prior to blocking.

#### PAR dot-blot binding assays

1 μg and 2 μg protein were spotted on nitrocellulose membrane for PAR binding and Ponceau S staining respectively. Protein dilutions were prepared in 200 μL TBS buffer (pH 7.5). Proteins were spotted using the Bio-Dot microfiltration Apparatus (Bio-Rad). Nitrocellulose membrane spotted with protein were incubated with Ponceau S stain (Sigma) for 5 mins followed by several quick washes with ultra-pure water to remove excess stain. For radioactive *in vitro* PAR binding assays, BLOTTO-blocked membranes with spotted proteins were incubated with radioactively-labeled PAR for 1hr at room temperature in TBS-T. Membranes were subsequently washed with TBS-T and TBS-T containing 1M NaCl. Washed and dried membranes were exposed to Hyperfilm MP (GE Heathcare). Radioactive PAR was synthesized in 50 μL reactions containing 10 μL of 5x PAR synthesis buffer (1x buffer = 25mM Tris-Cl ph.8, 0.1M NaCl, 10mM MgCl_2_ and 0.5mM DTT), 2 μL (20U) of recombinant human PARP-1 enzyme (Trevigen or SigmaAldrich), 2.5 μg calf thymus DNA (Invitrogen), 1 μL of 50mM NAD and 5 μL of ^32^P-labeled NAD (800 Ci/mmol; Hartmann Analytic) for 30min at 25°C. PAR chains were detached form PARP-1 by sequential addition to these reactions of DNase and proteinase K (NEB) followed by heat inactivation.

#### EMSA/Gel shift assay

The DNA oligos (NBRE: 5′-GATCCTCGTGCGAAAAGGTCAAGCGCTA-3′ and 5′-GAGCACGCTTTTCCAGTTCGCGATCTAG-3′; mutNBRE: 5′-GATCCTCGTGCGAAAAGGTCAAGCGCTA-3′ and 5′-GAGCACGCTTTTCCAGTTCGCGATCTAG-3′) were annealed by combining two single strand oligos in TE buffer pH 7.5 and 50mM NaCl, heating at 80°C for 15mins and then gradually cooling to room temperature. 2pmol annealed oligo was radioactively labeled using 10 μCi/μl α^32^P-dCTP (PerkinElmer), 0.1mM dNTPs (without dCTP), 5U Klenow fragment (NEB Inc) and 1X NEB 2 buffer incubated for 20mins at room temperature. The probe was further subjected to illustra ProbeQuant G-50 micro column (GE Healthcare) purificatioin to remove excess of unincorporated α^32^P-dCTP. Gel shift reaction mixture contained 1 μg protein, binding buffer (10mM Tris pH 8, 50mM NaCl, 1mM EDTA, 5% glycerol and 5mM DTT), 20ng/μl competitor dIdC (SigmaAldrich), 1.25mg/ml BSA and radioactive labeled oligos (NBRE or mutNBRE). The reaction was incubated for 20mins at room temperature. Loading dye (bromophenol blue 0.25%, xylene cyanol 0.2% and Ficoll 15%) was then added to the reaction mixture. The reaction mixture was loaded on 5% polyacrylamide gel using 0.25X TBE as running buffer. Prior to loading of the samples, the gel was pre-run for 15-30mins at 150V. The gel was then dried using Bio-Rad gel dryer system at 80°C and subjected to autoradiography.

#### Reporter gene assays

NuRE or NBRE based reporter gene was constructed by cloning four copies of NuRE (Philips et al., 1997) or NBRE response element in front of TK minimal promoter in pGL3 basic vector driving the expression of firefly reporter gene (Promega). Reporter gene assays were performed by co-transfection the reporter plasmid, NR4A2 expression plasmid and LacZ expression vector into U2OS cells in 24 well format in triplicates. After 24hr of incubation cells were lysed in 150 μL of passive lysis buffer (Promega). Luciferase assays were performed with commercial kit (cat. 16177; ThermoFisher Scientific) according to manufacturer’s manual and normalized to galactosidase activity (cat. T1007; Galacto-Light Plus β-Galactosidase Reporter Gene Assay System; ThermoFisher Scientific). Luciferase and galactosidase activity was measured in 96 well white plates read by multi-plate reader VictorX4 (Perkin Elmer).

#### CRISPR-Cas9-mediated deletion of NR4A1 and NR4A2

U2OS cells were transfected with a CMV promoter-driven Cas9-Puro expression vector and predesigned guide RNAs (gRNA) (Dharmacon) for each targeted gene using Lipofectamine 2000. After 24 hr at 37°C, puromycin (4 μg/ml) was added for an additional 24 hr to select for Cas9-expressing cells, and subsequently replaced with DMEM in the absence of puromycin for a further 48 hr. Thereafter cells were plated at a density of 500 cells per 10 cm dish or plated in 96-well plates at a density of 12.5 cells/ml. After 10-12 days, individual colonies were transferred to 12-well plates using trypsin/EDTA-soaked cloning discs, expanded and subsequently analyzed by immunoblotting for expression of NR4A1 and NR4A2. Identification of CRISPR-Cas9-mediated mutations (indels) near gRNA target sequences (Figure S3C) was performed by amplification of 0.4-1kb genomic DNA regions spanning gRNA target sequences (near PAM/cleavage sites) using Q5 high fidelity DNA polymerase. PCR products were ligated into pJET1.2 and DNA sequencing performed on plasmids recovered from bacteria for each independent clonal knockout cell line.

#### Molecular modeling

Figures 2A and 2H were prepared using PyMOL Molecular Graphic system, version 1.6.0.0 (Schrödinger, LLC). Crystal structure of NR4A1/NGFI-B DBD with DNA (PDB: 1CIT; (Meinke and Sigler, 1999)) was used to create Figure 2A. MetaPocket 2.0 online server (Ref: https://academic.oup.com/bioinformatics/article/27/15/2083/402380 and https://www.liebertpub.com/doi/abs/10.1089/omi.2009.0045) was used to predict ligand binding pocket on protein surface shown in Figure 2H. Crystal structure of DBDs from NR4A1/NGF1-B and NR5A2 (PDB: 1CIT and PDB: 2A66 respectively) and solution structure of NR5A1 (PDB: 2FF0) were used for ligand pocket prediction (Figure 2H). Crystal structure of NR4A1/NGFI-B DBD with DNA (PDB: ICIT; (Meinke and Sigler, 1999) and solution structure of first PBZ domain of APLF in complex with RFA were used to create Figure S2G (PDB: 2KQD; (Eustermann et al., 2010)). The online server MetaPocket 2.0 (Huang, 2009) was used to predict the ligand binding pocket for NR4A1 structure. MetaPocket is a consensus based method that combines results from four predictor methods: LIGSITEcs, PASS, Q-SiteFinder, and SURFNET to provide an improved prediction success rate. In short, the server verifies result from each predictor site, calculates a z-score for each prediction, obtains tops 3 pocket sites from each predictor and clusters them according to spatial similarity. Thereafter the algorithm ranks the clusters based on their z-score and finally calculates a mass center for each cluster further providing the user with an output information of prediction pocket site as single point or clusters. MetaPocket 2.0 as an online server works on the user-supplied structure information of protein of interest to find potential ligand binding pockets. For this we initially used two approaches; (i) we used the NR4A1/NGFI-B DBD with DNA (PDB: 1CIT) directly and (ii) we removed the DNA structure from the 1CIT structure and used this as a starting structure to predict pocket. Based on the calculations used by the server, using the PBD structure directly with DNA only predicted cluster pockets within the DNA. Therefore, in the second approach, when DNA molecule was removed from structure, the top pocket predictions were – (i) region of protein that we know from NR4A1/NGFI-B DBD structure to directly interact with DNA and (ii) a more globular shaped pocket that was predicted away from DNA binding site in the Zn2 domain of NR4A2, which we show in Figure 2H. Using the same web server, we also performed a ligand pocket prediction for NR5A receptor (PDB: 2A66) and NR5A1 receptor (PDB: 2FF0) proteins without DNA molecule.

#### Immunofluorescence

Cells were transfected with pCMX-NR4A2 encoding plasmids and at 24hr post-transfection were seeded on 8-well cluster chamber slides at 0.05x10^6^/well. After overnight incubation cells were fixed with 4% PFA at RT for 10min. Cells were washed with PBS and then incubated in blocking/permeabilisation solution (5% normal goat serum, PBS and 0.03% NP-40) at 4°C for 1hr. Primary antibodies were applied in blocking solution and slides were incubated overnight at 4°C. After subsequent PBS wash, secondary antibodies (Jackson Immunoresearch) were applied in blocking solution and slides were incubated 1hr at RT. Images were acquired on Zeiss LSM 510 confocal microscope.

#### Transfections

Cells were transiently transfected according to manufacturer’s instructions. For plasmid and siRNA/plasmid the Lipofectamine 2000 (U2OS) or LTX/Plus (293HEK) was used. For siRNA transfections Lipofectamine RNAiMAX was used.

#### Cell fractionation

Subcellular fractionation was performed with commercial kit (Product 78840, ThermoFisher Scientific) according to manufacturer’s manual. Each time point corresponds to subconfluent 6cm dish of ca 1-2 × 10^6^ cells.

#### Measuring HR and NHEJ efficiency with reporter cassettes

HR (pDRGFP plasmid; Addgene; (Pierce et al., 1999)) and NHEJ (pimEJ5GFP; Addgene; (Bennardo et al., 2008)) reporter plasmids were stably integrated into U2OS (Parental) or cA1/A2 cells by transient transfection followed by selection of stable cell lines with puromycin. For the measurement of the HR and NHEJ efficiency cells were transiently transfected in triplicate with I-SceI expression vector (gift from dr Benjamin Chen, UTSouthwestern). After 24hr of incubation GFP positive cells were quantified by FACS. Transfection efficiency was monitored by RFP co-expression. Efficiency of HR and NHEJ in cA1/A2 cells was calculated as percentage of efficiency observed in Parental cells that was set to 100%.

#### Laser Micro-irradiation and Live Cell Imaging

U2OS cell were transiently transfected with EGFP-Ku70 or EGFP-N1-NR4A2 expression plasmids with the use of Lipofectamine 2000. After 48 hr cells were presensitized with Hoechst 33342 (10 μM) immediately prior to live cell imaging. Live cell time lapse imaging combining laser micro-irradiation with confocal microscopy was performed by capturing images on a Marianas-SDC system from Intelligent Imaging Innovations (3i). This system uses a Yokogawa CSU-W with 150 mW 488nm excitation and Hamamatsu Flash 4.0 v2+ camera. DNA damage was introduced using the 3i ‘Ablate’ UV 355 nm pulsed laser system (70 μJ per pulse at 200Hz) which is focused to a diffraction limited spot at the sample plane and steered along a user-defined line by the 3i ‘VectorM’ MEMS mirror scanner and Slidebook software. Relative intensity at laser-damaged sites was calculated as the mean value of the intensity of each damage site at each time point after background subtraction. At least 10 cells were scored per sample and per experiment. Images presented in Figure 1A were generated according to (Mehrotra et al., 2011). In some experiments DMSO (Sigma-Aldrich) or 10 μm PARP inhibitor (PARPi; olaparib; purchased from SantaCruz biotechnology) was used to pre-treat cells 15-30min prior to laser irradiation.

#### DSB foci counting

Cells were fixed in PFA and stained with anti-53BP1 antibodies and DAPI (for RAD51 foci detection cells were fixed with methanol). Images of nuclei were acquired with high magnification to visualize individual 53BP1 nuclear foci. Individual foci were counted and the data was presented as mean foci per nucleus value. At least 30 cells were counted per sample per experiment.

#### Clonogenic survival assays

U2OS (Parental) or cA1/A2 cells were plated in triplicate at densities of 1000–30000 cells per 10 cm dish and exposed to X-Rays (0–4 Gy) and grown for 10–14 days to form colonies. Colonies were fixed in 75% methanol/25% acetic acid, prior to staining with PBS/0.05% (w/v) crystal violet and counting. The survival fraction was determined from the plating efficiency of the specific IR dose relative to the plating efficiency of non-irradiated controls.

#### Identification of C8ORF4/TC-1gene as NR4A2 target

U2OS cells were transiently transfected with pCXM-LacZ (mock control), pCMX-NR4A2 WT, pCMX-NR4A2 K303A or pCMX-NR4A2 R319A expression plasmids. After 16hr cells were harvested and total RNA was extracted (QIAGEN RNAeasy kit) and hybridized to 60K whole human genome microarrays (Agilent Technologies, Berkshire, UK), following the manufacturer’s protocol. RNA was first checked for quality using a 2100 Bioanalyser (Agilent). RNA with a RIN score of > 9 was used for microarray analysis. 200 ng RNA was Cy-3 labeled using Agilent Low Input Quick Amp 1-color Labeling Kit (Agilent). The level of dye incorporation was measured using a Nanodrop ND1000 spectrophotometer (LabTech, Sussex, UK). 600 ng Cy-3 labeled sample was fragmented using fragmentation buffer from the Agilent Gene Expression Hybridization Kit (Agilent) for 30 min at 60°C. Fragmented samples were mixed 1:1 with Hybridization Buffer (v/v) and hybridized to a 60K high-density oligonucleotide microarray overnight. Microarrays were loaded as per the manufacturer’s instructions and hybridization was performed at 65°C, in an Agilent Hybridization Oven with rotation set to 10 RPM for 17 h. Following hybridization, the microarray slides were washed in Gene Expression wash buffers 1 and 2 (Agilent) and immediately scanned using a DNA Microarray Scanner (Model G2505C, Agilent Technologies) at 3 μm resolution. Scanned images were uploaded into Agilent’s Feature Extraction software (v. 10.7.3.1). Within array normalization, background subtraction and flagging of outliers due to saturation or non-uniformity was performed. Processed signal data for each probe was extracted for further analysis. Extracted data was analyzed using GeneSpring GX 12.6.1. Data was normalized to the 75^th^ percentile and then subjected to a moderated one-way ANOVA test. Genes were declared as differentially regulated if they showed a fold change of ≥ 2 with corrected *p* value ≤ 0.05 after Benjamini Hochberg based False Discovery Rate correction. This led to identification of *C8ORF4/TC-*1gene as NR4A2 responsive target (Table S1). *C8ORF4/TC-*1upregulation after transfection of NR4A2 was verified by qPCR as follows: 16hrs after transfection RNA was extracted from cells using RNeasy kit (QIAGEN). Reverse transcription reaction was conducted with superscript II (ThermoFischer Scientific) using 1 μg of total RNA. qPCR was conducted using FAST SYBR green qPCRpremix (Applied Biosystems, ThermoFisher Scientific) and plates were analyzed on Quantstudio 6 flex (Applied Biosystems). QPCR results were quantified using delta delta ct method with *GAPDH* serving as reference.

### Quantification and Statistical Analysis

Statistical analysis was performed with GraphPad Prism software (v. 6). Statistical significance was assessed with paired two-tailed t test. Results were considered statistically significant when p value was lower than 0.05; SD represents standard deviation; SEM denotes standard error of the mean; n value represents the number of biological replicates.

## Supplemental Information

Document S1. Figures S1–S4 and Table S1

Document S2. Article plus Supplemental Information
