# Supplementary material for: NR4A Nuclear Receptors Target Poly-ADP-Ribosylated DNA-PKcs Protein to Promote DNA Repair
Source: Cell Rep. 2019 Feb 19;26(8):2028–2036.e6. doi: 10.1016/j.celrep.2019.01.083 (PMC6381605; doi:10.1016/j.celrep.2019.01.083)
Supplement: Document S1. Figures S1–S4 and Table S1 [file mmc1.pdf]

Cell Reports, Volume *26*

Supplemental Information

NR4A Nuclear Receptors Target Poly-ADP-Ribosylated DNA-PKcs Protein to Promote DNA Repair

Deeksha Munnur, Joanna Somers, George Skalka, Ria Weston, Rebekah Jukes-Jones, Mohammed Bhogadia, Cyril Dominguez, Kelvin Cain, Ivan Ahel, and Michal Malewicz

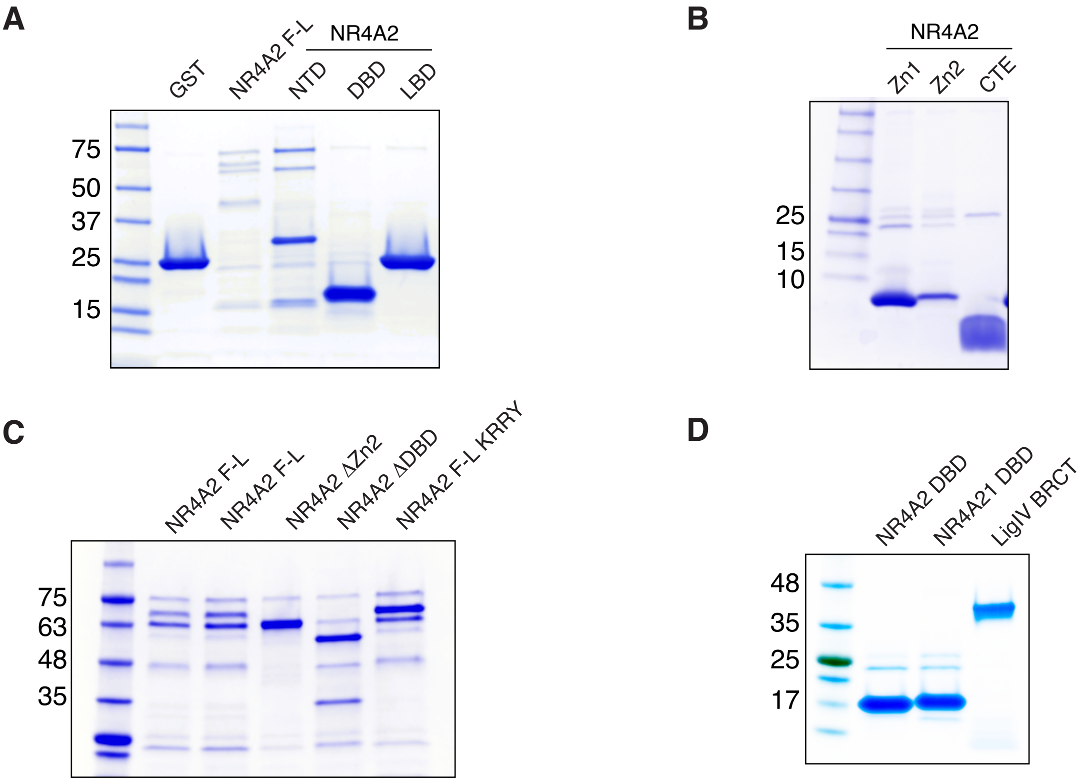

E

**NR4A2**	FKRTVQKNAKYVCLANKNCPVDKRRRNRCQYC**R**FQKC	323
**NR4A1**	FKRTVQKNAKYICLANKDCPVDKRRRNRCQFC**R**FQKC	
**NR4A3**	FKRTVQKNAKYVCLANKNCPVDKRRRNRCQYC**R**FQKC	
**DHR38**	FKRTVQKGSKYVCLAEKSCPVDKRRRNRCQFC**R**FQKC	
**NR5A1**	FKRTVQNNKHYTCTESQSCKIDKTQRKRCPFC**R**FQKC	
**NR5A2**	FKRTVQNQKRYTCIENQNCQIDKTQRKRCPYC**R**FKKC	
**DHR39**	FKRTVQNRKNYVCVRGGPCQVSISTRKKCPAC**R**FEKC	
**RXRα**	FKRTVRKDLTYTCRDNKDCLIDKRQRNRCQYC**R**YQKC	
**RXRβ**	FKRTIRKDLTYSCRDNKDCTVDKRQRNRCQYC**R**YQKC	
**RXRγ**	FKRTIRKDLIYTCRDNKDCLIDKRQRNRCQYC**R**YQKC	
**USP**	FKRTVRKDLTYACRENRNCIIDKRQRNRCQYC**R**YQKC	
**CAR**	FRRTVSKSIGPTCPFAGSCEVSKTQRRHCPAC**R**LQKC	
**RARα**	FRRSIQKNMVYTCHRDKNCIINKVTRNRCQYC**R**LQKC	
**ERRα**	FKRTIQGSIEYSCPASNECEITKRRRKACQAC**R**FTKC	
**ERRβ**	FKRTIQGNIEYSCPATNECEITKRRRKSCQAC**R**FMKC	
**GR**	FKRAVEGQHNYLCAGRNDCIIDKIRRKNCPAC**R**YRKC	
**ERβ**	FKRSIQGHNDYICPATNQCTIDKNRRKSCQAC**R**	
**AR**	FKRAAEGKQKYLCASRNDCTIDKFRRKNCPSC**R**	
**PR**	FKRAMEGQHNYLCAGRNDCIVDKIRRKNCPAC**R**	
**COUPTF2**	FKRSVRRNLSYTCRANRNCPIDQHHRNQCQYC**R**LKKC	
**HNF4α**	FRRSVRKNHMYSCRFSRQCVVDKDKRNQCRYC**R**LKK	
**PPARγ**	FRRTIRLKLIY-DRCDLNCRIHKKSRNKCQYC**R**FQKF	

Figure S1. Related to Figure 1.
A), B), C), D) Coomassie stain of indicated bacterially expressed and purified proteins. Mw protein markers are shown on the left. E) Alignment of the main mammalian nuclear receptor's (NRs) Zn2 domains. Top panel groups NR4A family including DHR38 (Drosophila NR4A homologue). Red indicates residues involved in NR4A PAR-binding and their relative conservation across different nuclear receptors. Bold underlined is the R319 residue of NR4A2 and corresponding residues in other nuclear receptors. Note the invariant conservation of R319 across the whole nuclear receptor superfamily.

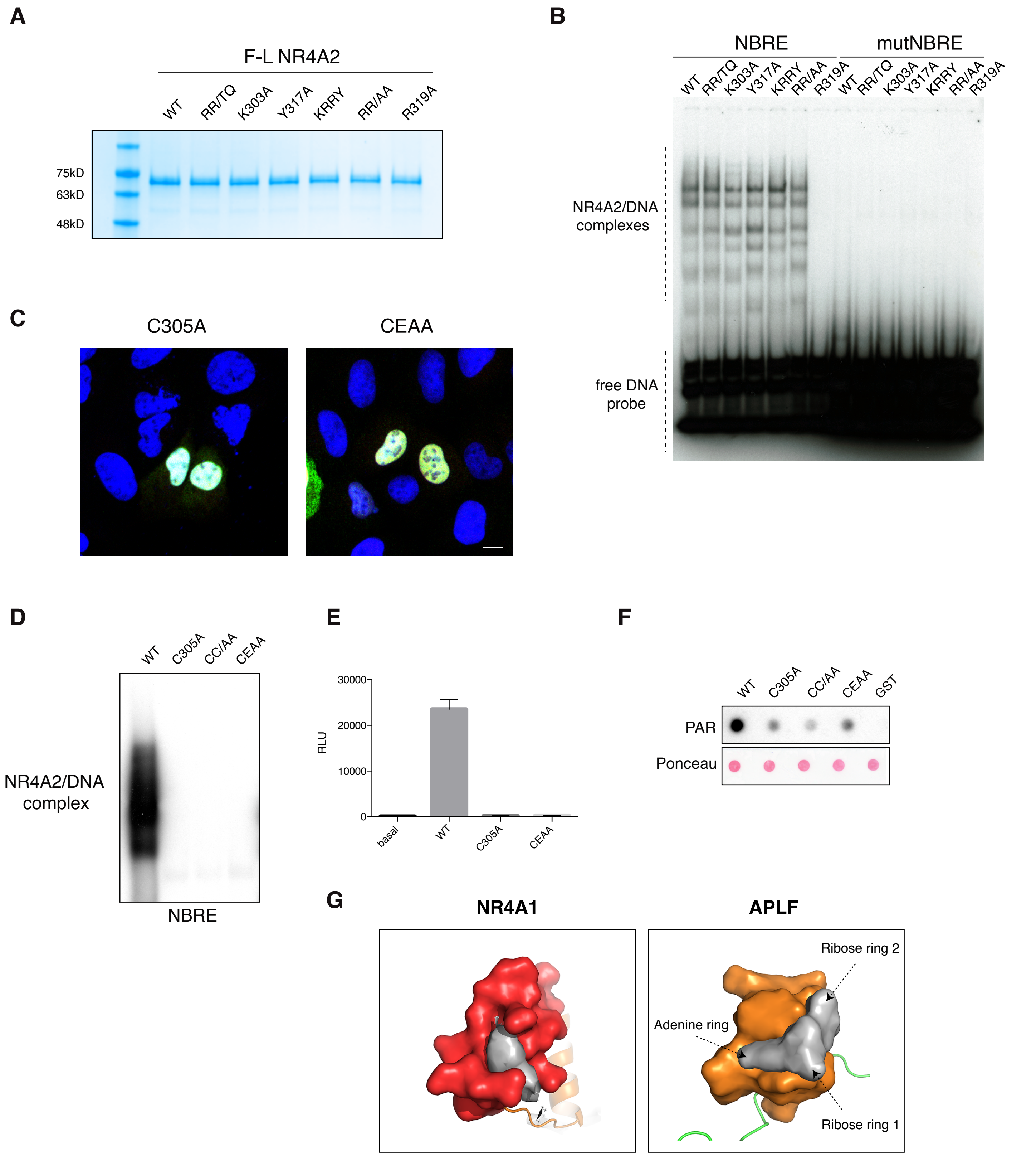

Figure S2. Related to Figure 2.
A) Coomassie stain of indicated bacterially expressed and purified proteins after gel filtration column. B) Full gel size image of the EMSA assay shown in Fig. 2C. C) U2OS were transiently transfected with plasmids expressing indicated NR4A2 mutants. Representative immunofluorescence images showing DAPI nuclear staining (blue) superimposed with NR4A2 signal (green). Scale bar = 10μm. D) EMSA sequence-specific DNA binding assay with indicated bacterially expressed full-length NR4A2 proteins and NBRE DNA oligo probe. E) NBRE-based reporter gene assays with indicated NR4A2 proteins. (Basal) indicates activity of LacZ transfected mock control sample. Graph shows mean relative luminesce (RLU) with S.D. plotted (n=3). F) Dot-blot PAR-binding assay with indicated bacterially expressed NR4A2 proteins. Ponceau stain serves as loading control. G) Comparison of the PAR binding pocket of NR4A1 (as predicted by MetaPocket server) with the fi¬rst zinc ¬finger domain of APLF bound to ribofuranosyladenosine (RFA) ligand. In the APLF structure, the adenine ring of RFA is stacked between two tyrosine residues of the zinc finger of APLF, which forms a narrow cleft like pocket on the protein surface and the two ribose rings twist out of plane. Compared to the RFA structure the NR4A1 binding site predicted by MetaPocket is smaller and more globular in shape. However, the binding surface of the NR4A1 also has wider cleft-like fold, which could accommodate PAR chains similar to APLF. While the cluster of binding site predicted for NR4A1 does not exactly match the RFA structure, the overall protein fold around the cluster/ligand appears to be well ordered envelope that could encompass the bound PAR chain.

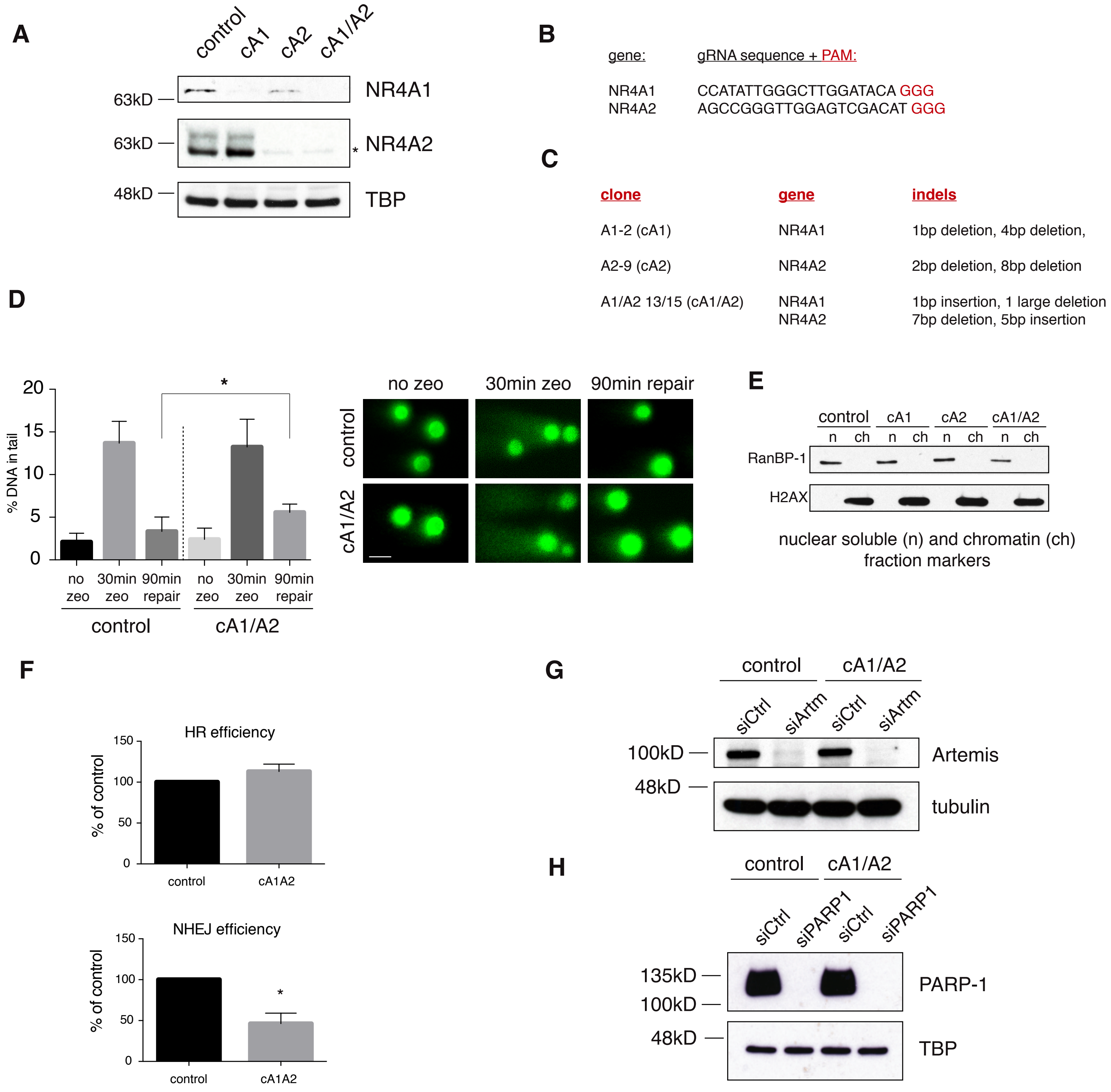

Figure S3. Related to Figure 3.
A) Western blotting confirmation of CRISPR-based deletion of NR4A1 and NR4A2 in U2OS nuclear extracts. Control indicates reference parental U2OS cells; cA1 indicates NR4A1 knockout clone; cA2 indicates NR4A2 knockout clone; cA1/A2 indicates NR4A1/A2 double knockout clone. TBP is used as loading control. Mw protein markers are shown on the left. (*) indicates a non-specific band migrating slightly above Tinur. B) Sequence of synthetic RNA guides used in CRISPR targeting experiments. PAM sequence is shown in red. C) Structure of indels found in the vicinity of PAM complementary sequence in individual NR4A1/2 knockout clones as revealed by sequencing. D) Neutral COMET assays detecting DSBs in parental (control) and cA1A2 cells. Graph presents the quantification of the percentage (%) of DNA in COMET tail at indicated time points. (no zeo) control sample; (zeo 30min) sample treated for 30min with zeocin prior to harvest; (90 min repair) sample treated with zeocin for 30min followed by zeocin washout and further 90min incubation to allow for DSB repair. S.D. plotted above individual data points (n=3). Asterisk denotes a statistically significant difference in relation to indicated reference sample (p value<0.05). Right panel shows representative COMET images. Scale bar = 20µm. E) Demonstration of purity of nuclear soluble (n) and chromatin fractions (ch) used in experiments shown in Figure 3E/F using relevant marker proteins as indicated. F) HR (upper panel) and NHEJ (lower panel) reporter gene assays in parental (control) or cA1A2 cells (n=3; error bars represent S.D.). Asterisk denotes a statistically significant difference in relation to control sample (p value<0.05). G) Western blot verification of Artemis knockdown efficiency in whole cell extracts of control (U2OS) and cA1/A2 cells at 48hr after transfection with reference (siCtrl) or Artemis-specific (siArtm) siRNAs. Tubulin serves as a loading control. H) Western blot verification of PARP1 knockdown efficiency in whole cell extracts of control (U2OS) and cA1/A2 cells at 48hr after transfection with reference (siCtrl) or PARP1-specific (siPARP1) siRNAs. TBP serves as a loading control.

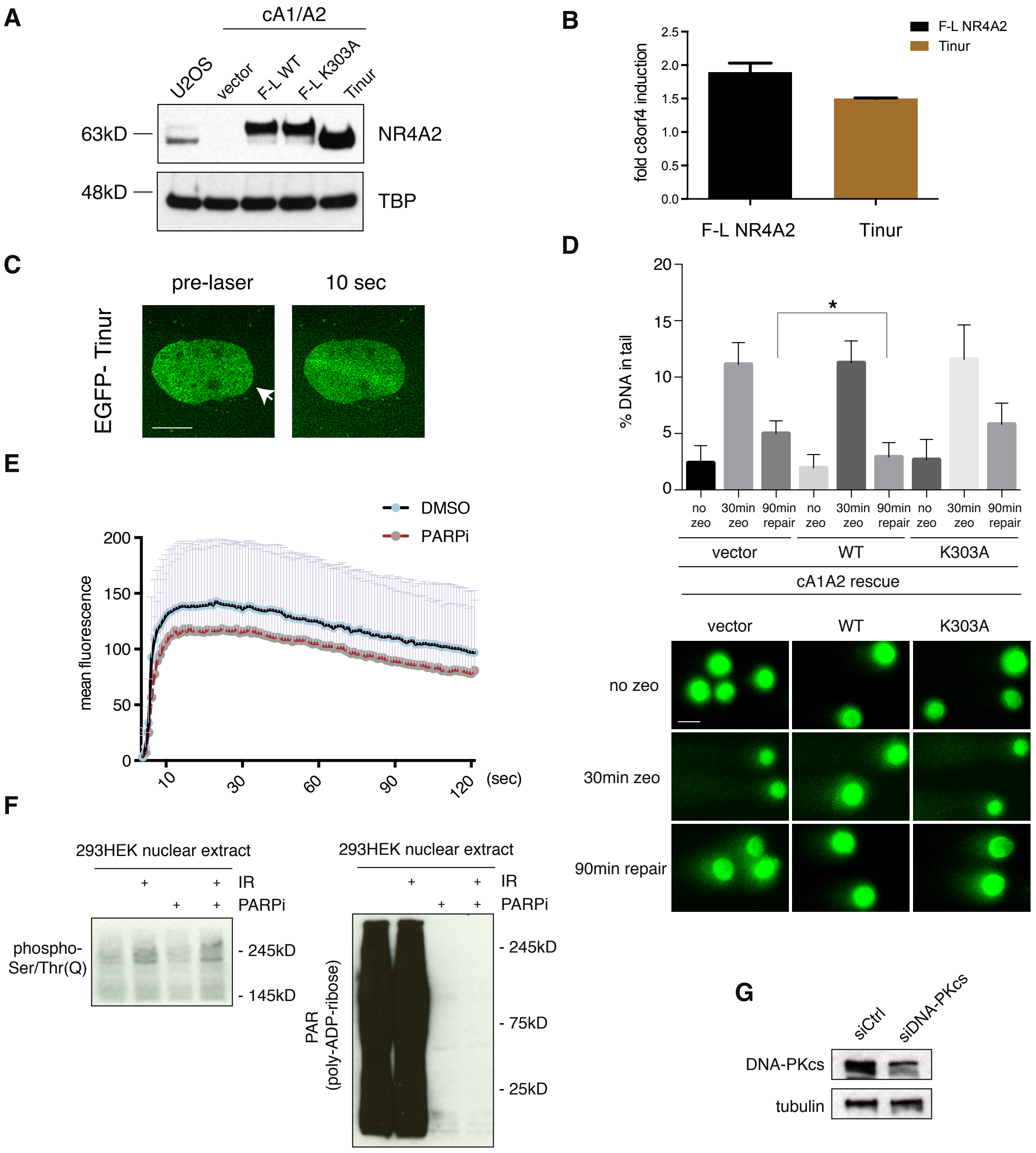

Figure S4. Related to Figure 4.
A) Western blot verification of re-expression of full-length (F-L) WT NR4A2, full-length (F-L) K303A NR4A2 and Tinur NR4A2 isoform in nuclear extracts from indicated cell lines. TBP is used as loading control. Mw protein markers are shown on the left. B) Transcriptional activity as fold induction of TC-1/c8orf4 mRNA measured by qPCR after transfection of either WT full-length (F-L) NR4A2 or Tinur isoform and normalisation to LacZ (mock) transfected sample. S.D. is plotted on the graph (n=3). C) Laser micro irradiation of U2OS cells transiently transfected with plasmid encoding EGFP-Tinur fusion protein. Arrow shows the irradiated position in the nucleus. Scale bar = 10µm. D) Neutral COMET assays detecting DSBs in cA1A2 cells rescued with vector (vector), WT F-L NR4A2 (WT) or K303 F-L NR4A2 (K303A) expressing viruses. Graph presents the quantification of the percentage (%) of DNA in COMET tail at indicated time points. (no zeo) control sample; (zeo 30min) sample treated for 30min with zeocin prior to harvest; (90 min repair) sample treated with zeocin for 30min followed by zeocin washout and further 90min incubation to allow for DSB repair. S.D. plotted above individual data points (n=3). Asterisk denotes a statistically significant difference in relation to indicated reference sample (p value<0.05). Lower panel shows representative COMET images. Scale bar = 20µm. E) Quantification of the EGFP-Ku70 recruitment to DSBs by laser micro irradiation before and after PARP inhibitor (PARPi) treatment. DMSO indicates vehicle treated sample. Graph presents the quantification of laser micro irradiation experiments (n=3) with S.D. plotted above individual data points. F) Western blotting of nuclear extracts from 293 cells treated with PARP inhibitor (PARPi; olaparib; 10µM) for 3hr plus/minus ionizing radiation (IR; 20Gy). Left panel shows signal of anti-phospho-Ser/Thr(Q) PIKK kinase substrate specific antibody (IR positive control), right panel shows signal of anti-poly-ADP-ribose (PAR) specific antibody (control for PARPi activity). G) Western blotting confirmation of DNA-PKcs protein depletion with siRNA. U2OS cells were transfected with control (siCtrl) or DNA-PKcs-specific (siDNA-PKcs) siRNA, incubated for 48hr prior to WCE extraction. Tubulin is used as loading control.

Michal Malewicz microarray analysis
Notes : Fold change **>= 2.0**
#Entity List : **Oneway ANOVA p (Corr) cut-off = 0.05**
#Condition pairs :
#[K303A] vs [LacZ]
#[R319A] vs [LacZ]
#[WT] vs [LacZ]
#Minimum number of pairs:1 out of 3 condition pairs.
Technology : Agilent.SingleColor.39494
Owner : gxuser
Created On : Tue Aug 09 14:19:47 BST 2016

**GeneSymbol**	**EntrezGeneID**	**ProbeName**	**K303A vs LacZ**			**R319A vs LacZ**			**WT vs LacZ**			**Description**	**Sequence**
			**FC**	**Log FC**	**Regulation**	**FC**	**Log FC**	**Regulation**	**FC**	**Log FC**	**Regulation**		
NR4A2	4929	A_33_P3299066	236.66	7.89	up	316.30	8.31	up	267.80	8.07	up	Homo sapiens nuclear receptor subfamily 4, group A, member 2 (NR4A2), mRNA [NM_006186]	GACTTGGTGCCACCGCCAGCAATAATTGACAAACTTTTCCTGGACACTTTACCTTTCTAA
NR4A2	4929	A_23_P131208	39.64	5.31	up	49.63	5.63	up	43.79	5.45	up	Homo sapiens nuclear receptor subfamily 4, group A, member 2 (NR4A2), mRNA [NM_006186]	AAGTTTTCTGCTGTAAAGAAAGCTGTAATATATAGTAAAACTAAATGTTGCGTGGGTGGC
C8orf4	56892	A_23_P253350	3.99	2.00	up	1.17	0.22	up	4.01	2.00	up	Homo sapiens chromosome 8 open reading frame 4 (C8orf4), mRNA [NM_020130]	TGAAAAAGTGCACCACATGGATGTTAAGTAGAAATTCAAGAAAGTAAGATGTCTTCAGCA

Table S1. Overexpression of NR4A2 upregulates the *C8ORF4* mRNA. **Related to Figure 2.**
